# Alpha cell receptor for advanced glycation end products associate with glucagon expression in type 1 diabetes

**DOI:** 10.1038/s41598-023-39243-x

**Published:** 2023-08-09

**Authors:** Sherman S. Leung, Nataliya Lenchik, Clayton Mathews, Alberto Pugliese, Domenica A. McCarthy, Selena Le Bagge, Adam Ewing, Mark Harris, Kristen J. Radford, Danielle J. Borg, Ivan Gerling, Josephine M. Forbes

**Affiliations:** 1 Glycation and Diabetes Complications, Mater Research Institute, Translational Research Institute (TRI), The University of Queensland (MRI-UQ), 37 Kent Street, Woolloongabba, Brisbane, QLD 4102 Australia; 2 Faculty of Medicine, The University of Queensland, Brisbane, Australia; 3 School of Medicine and Dentistry, Griffith University, Brisbane, Australia; 4 Wesley Research Institute, The Wesley Hospital, Brisbane, Australia; 5 Division of Endocrinology, Department of Medicine, University of Tennessee Health Science Center, Memphis, TN USA; 6 Division of Endocrinology, Department of Microbiology and Immunology, Department of Medicine, Diabetes Research Institute, Miller School of Medicine, University of Miami, Miami, FL USA; 7 Translational Bioinformatics Group, MRI-UQ, TRI, Brisbane, Australia; 8 Queensland Diabetes Centre, Mater Health Services, Brisbane, Australia; 9 Cancer Immunotherapies Group, MRI-UQ, TRI, Brisbane, Australia

**Keywords:** Diabetes complications, Type 1 diabetes, Paediatric research, Endocrine system and metabolic diseases

## Abstract

Hypoglycemia in type 1 diabetes associates with changes in the pancreatic islet α cells, where the receptor for advanced glycation end products (RAGE) is highly expressed. This study compared islet RAGE expression in donors without diabetes, those at risk of, and those with type 1 diabetes. Laser-dissected islets were subject to RNA bioinformatics and adjacent pancreatic tissue were assessed by confocal microscopy. We found that islets from type 1 diabetes donors had differential expression of the RAGE gene (*AGER*) and its correlated genes, based on glucagon expression. Random forest machine learning revealed that *AGER* was the most important predictor for islet glucagon levels. Conversely, a generalized linear model identified that glucagon expression could be predicted by expression of RAGE signaling molecules, its ligands and enzymes that create or clear RAGE ligands. Confocal imaging co-localized RAGE, its ligands and signaling molecules to the α cells. Half of the type 1 diabetes cohort comprised of adolescents and a patient with history of hypoglycemia—all showed an inverse relationship between glucagon and RAGE. These data confirm an association between glucagon and islet RAGE, its ligands and signaling pathways in type 1 diabetes, which warrants functional investigation into a role for RAGE in hypoglycemia.

## Introduction

Hypoglycemia in type 1 diabetes is attributed to a dysfunction of the pancreatic islet α cells and the autonomic counterregulatory response. The dysfunction of the α cells is characterized by excessive basal secretion of glucagon, termed “hyperglucagonemia”, and insufficient glucagon secretion in response to decreasing blood glucose concentrations, which contributes to hypoglycemic events^1^. Severe hypoglycemia requiring medical assistance affects 30–40% of individuals with type 1 diabetes, with individuals typically having at least one hypoglycemic episode per year^2^. Hypoglycemia increases the risk of cognitive dysfunction^3^ and all-cause mortality^4^, and is associated with neuronal cell death^5–7^.

The “switch-off” hypothesis is the leading model for dysfunctional glucagon responsiveness in type 1 diabetes^8,9^. This theory suggests that the cessation of insulin secretion from β cells in response to hypoglycemia, is a critical trigger for the rapid release of glucagon to raise blood glucose concentrations by gluconeogenesis. Hence, the absence of insulin-producing β cells in type 1 diabetes is postulated to impair the rapid triggering of glucagon secretion from α cells. However, recent studies using human pancreatic samples acquired from organ donors by the Network for Pancreatic Organ Donors with Diabetes (nPOD)^10^ have found intrinsic aberrations in the α cells per se in type 1 diabetes, which challenges a β cell focused "switch-off" hypothesis.

These studies identified a specific decrease in α cell glucagon (*GCG*) gene expression and secretion in recent-onset type 1 diabetes^11^. There were also reports of decreased expression of a glucagon inhibitory protein in α cells from donors with type 1 diabetes^12^. Human islet studies have also demonstrated reduced α cell mass^13^, as well as proliferative^14^ and immunological changes in the α cells in type 1 diabetes^15^. These data allude to intrinsic differences in the α cells that may contribute to resting hyperglucagonemia and a dysfunctional glucagon response in type 1 diabetes. Indeed, novel pathways that can be targeted to reduce the risk of hypoglycemia are of clinical interest, as recently highlighted by favorable Phase 1b/2 data of a hepatoselective glucokinase activator (NCT03335371).

The receptor for advanced glycation end products (RAGE) is a pattern recognition receptor expressed in several tissues including the pancreatic islets^16,17^. Increased islet RAGE expression has been reported in both type 1 and type 2 diabetes^18,19^. Interestingly, RAGE expression is most prominent in α cells^17,18^, binds to a range of ligands including high mobility group box protein 1 (HMGB1)^16^ and can be targeted to prevent the onset of type 1 diabetes in preclinical models^20^. RAGE ligation also elicits various signaling cascades implicated in the pathogenesis of type 1 diabetes including the Janus kinases (JAK), signal transducer and activator of transcription proteins (STAT), nuclear factor-κB (NF-κB) and mitogen-activated protein kinases (MAPK)^16^. It remains unclear if modulation of RAGE can directly impact α cell function.

Here, we examined laser-captured islets from pancreata collected by nPOD from donors classified as (i) controls (without diabetes), (ii) at risk of type 1 diabetes (autoantibody-positive) or (iii) diagnosed with type 1 diabetes, to investigate islet RAGE expression in α cells. Bioinformatic analyses focused on genes where expression correlated with the expression of the RAGE gene (*AGER*), identifying an association between *AGER* and *GCG* expression using numerous modelling and comparison methodologies. These findings were confirmed with quantitative confocal imaging of RAGE, its ligands and signaling pathways, which were colocalized within the α cells. Functional evaluation for a role of RAGE and its ligands in glucagon secretion and expression in type 1 diabetes is warranted given the associations identified here.

## Materials and methods

### Pancreatic donors and tissue acquisition

Pancreata were procured from JDRF nPOD where tissues were recovered from organ donors as previously described^10^. Procedures were approved by the University of Florida Institutional Review Board (201400486) and the United Network for Organ Sharing (UNOS) according to federal guidelines and the Declaration of Helsinki. Informed consent was provided by each donor’s legal representative. Autoantibody status^21^ and C-peptide concentrations^10^ were determined as previously described^21^ and type 1 diabetes was diagnosed according to the American Diabetes Association’s guidelines. Data for other clinical characteristics were obtained from hospital records or UNOS. Approval for this study was provided by the nPOD Tissue Prioritization Committee, the Mater Human Research Ethics Committee (HREC/16/MHS/70) and The University of Queensland (2016/HE001566).

### RNA extraction and microarray

Human pancreatic cryosections were obtained from nPOD for the isolation of islets by laser-capture microscopy. Following the pooling of 20–30 islets/donor, RNA was isolated using the Arcturus PicoPure RNA Isolation Kit (Applied Biosystems; KIT0204), amplified by the WT-Ovation™ Pico RNA System (Integrated Sciences; 3302-12), and hybridized on the GeneChip™ Human Gene 2.0 ST microarray (Applied Biosystems)^22^.

### Bioinformatics

Raw intensities from the microarray were robust multichip average corrected, quantile normalized, median polish summarized and log2 transformed. To test the hypothesis that islet *AGER* associates with α cell function, a targeted analyses was performed on genes that correlated in expression with *AGER*^23^. To this end, *AGER* curated probes (17017764–17017785) were identified by annotations in Affymetrix Human Gene 2.0ST (Release 36). Genes correlated with any of the 22 *AGER* probes were subject to downstream analyses.

As the highest levels of RAGE expression were in the α cells, cohorts were stratified into *GCG*^hi^ or *GCG*^lo^ based on their intensity values for transcript cluster 16904315 (*GCG*). Using R v.3.2.2, volcano plots were created with *limma*, hierarchical clustering was created with *heatmap.2*, and modelling was performed with *glm* (further information is available in the Supplemental Methods), *car, sandwich* and *randomforest*. Graphs and data cleaning were performed with *broom*, *gplots* and *RColorBrewer*.

Gene Ontology enrichment was performed with the Gene Ontology database (Release 2017-06-29) and PANTHER Overrepresentation Test (Release 2017-04-13). Gene Set Enrichment Analysis (GSEA) was performed with GSEA v3.0 (Broad Institute, Cambridge, MA).

### Immunofluorescence

Formalin-fixed paraffin-embedded human pancreata were used for immunofluorescence analyses. To maintain a spatially consistent source of tissue, staining was performed in sections that were physically adjacent to the frozen tissue sections analyzed by microarray i.e., sections were separated by less than ~ 0.5 cm. Sections were deparaffinized in xylenes, rehydrated in graded ethanols and stained with primary antibodies against glucagon (T-5037, Peninsula Laboratories), insulin (MAB1417, RnD Systems), RAGE (AB5484, Millipore), the RAGE ligands N^∊^-(Carboxymethyl)lysine/CML (ab27684, Abcam) and HMGB1 (ab18256, Abcam), as well as the RAGE signaling molecules RELA (NF-κB p65; sc-8008, Santa Cruz), ERK2 (MAPK1, sc-136288, Santa Cruz) and JAK1 (3334, Cell Signaling). For the quantification of glucagon, insulin, RAGE and CML, antigen retrieval was not required, but for all other markers, antigen retrieval was performed by boiling in sodium citrate pH 6 for 20 min using a microwave. Multiplexed detection of primary antibodies was performed using anti-guinea pig DyLight800 (SA510100), anti-rat AlexaFluor488 (A21208), anti-goat AlexaFluor633 (A21082), anti-mouse AlexaFluor647 (A31571) and anti-rabbit AlexaFluor568 (A10042, ThermoFisher for all). Cross-reactivity was absent between primary antibodies and secondary antibodies produced against another host. Slides were mounted in Fluoroshield containing DAPI (F6057, Sigma-Aldrich) and visualized using the 60× oil-immersion lens (NA 1.35) on an Olympus FV1200 confocal laser scanning microscope.

Images were analyzed on ImageJ v2.0.0. Masking was performed for the localization of fluorescence intensities within glucagon-expressing α cells and insulin-expressing β cells. Multi-spectral overlays were created by merging individual frames for each channel, which were independently captured on the confocal microscope to eliminate cross-excitation and emission bleed through. Line scans were performed with the line profile tool.

### Quantification and statistical analysis

Statistical significance was defined as *p* < 0.05 in the Kruskal–Wallis and Dunn’s post-hoc test for multiple comparisons, *p* < 0.05 of Pearson’s *r* for correlation analysis, false discovery rate (FDR) adjusted *q* < 0.05 and fold-change > 2.0 for volcano plots, and Bonferroni adjusted *p* < 0.05 for Gene Ontology enrichment and over-representation tests. Data were analyzed in GraphPad Prism v.7.0.3 and R v.3.2.2 with the bioinformatic packages described above.

## Results

### Islet *GCG* expression is associated with increases in *AGER* and *AGER* correlated genes in type 1 diabetes

Donors with type 1 diabetes were younger, had lower C-peptide concentrations, elevations in HbA_1C_ and more autoantibodies, as compared with the control and “at risk” autoantibody-positive groups (Table 1). For all other clinical data, including HLA haplotypes, donor cohorts were matched (Table 1).

**Table 1:** Clinical characteristics of nPOD donors.

Characteristic	Control (Ctr, *n* = 18)	“At-risk” autoantibody-positive (Ab, *n* = 12)	Type 1 diabetes (T1D, *n* = 20)
Sex (number)			
Female	9	5	10
Male	9	7	10
Age (year)	36.0 ± 16.16	37.21 ± 18.24	19.97 ± 9.672*
BMI (kg/m^2^)	25.26 ± 4.817	24.89 ± 6.015	24.05 ± 4.249
Ethnicity (number)			
Caucasian	15	10	16
African American	2	1	4
Hispanic	1	1	0
Asian	0	0	0
Diabetes duration (years)	N/A	N/A	5 (6.815)
HbA_1c_ [% (mmol/mol)]	5.688 ± 0.372 (38.669)	5.425 ± 0.1708 (35.795)	11.12 ± 1.983 (98.041)*
C-peptide (ng/mL)	2.955 (3.89)	5.43 (11.71)	0.05 (0.05)***
Autoantibody positive (number of donors)			
GADA^+^ (IU/ml)	0	11	8*
IA2A^+^ (IU/ml)	0	2	9*
ZnT8A^+^ (IU/ml)	0	1	7*
mIAA^+^ (index)	0	2	14*
HLA haplotypes (number)			
DR3 allele	5	2	7
DR4 allele	4	5	4
DR3/4	2	0	5

HLA-DR3, HLA-DRB1*03; HLA-DR4, HLA-DRB1*04. Continuous variables that were normally distributed were compared by 1-way ANOVA and Tukey's post-hoc (shown as mean ± SD). Continuous variables that were non-normally distributed (diabetes duration, C-peptide) were compared by Kruskal–Wallis and Dunn's post-hoc [shown as median (IQR)]. Contingency table proportions were analyzed by Fisher's exact test. * *p* < 0.05 vs. all other groups; *** *p* < 0.001 vs. all other groups.

Laser-captured islets from donors were stratified into *GCG*^hi^ and *GCG*^lo^ expressing subgroups (Table 2) and their relationship to the RAGE gene, *AGER*, and its correlated genes were examined. When all donors were stratified in this manner, the proportion of control, non-diabetic autoantibody-positive and type 1 diabetes donors classified into the *GCG*^hi^ and *GCG*^lo^ subgroups was similar (Table 2). There were no differences in clinical characteristics between *GCG*^hi^ and *GCG*^lo^ subgroups, apart from a clinically insignificant elevation in HbA_1c_ in *GCG*^hi^ donors within the control cohort (vs. *GCG*^lo^ control donors; *p* < 0.05) (Table 2).

**Table 2:** Clinical characteristics of nPOD donors following stratification into *GCG*^*hi*^ or *GCG*^*lo*^ subgroups.

Characteristic	All donors (*n* = 50)		Control (Ctr, *n* = 18)		“At-risk” autoantibody-positive (Ab, *n* = 12)		Type 1 diabetes (T1D, *n* = 20)	
	*GCG*^*lo*^ (*n* = 25)	*GCG*^*hi*^ (n = 25)	*GCG*^*lo*^ (n = 9)	*GCG*^*hi*^ (*n* = 9)	*GCG*^*lo*^ (*n* = 6)	*GCG*^*hi*^ (*n* = 6)	*GCG*^*lo*^ (*n* = 10)	*GCG*^*h*^ ^*i*^(*n* = 10)
Cohort distribution (number; shown as Ctr, Ab, T1D)	10, 5, 10	8, 7, 10	–	–	–	–	–	–
Sex (number)								
Female	14	10	5	4	3	2	7	3
Male	11	15	4	5	3	4	3	7
Age (years)	26.4 ± 17.1	33.5 ± 15.2	30.5 ± 17.5	41.6 ± 13.4	41.5 ± 25.0	32.9 ± 8.0	16.7 ± 8.9	23.3 ± 9.7
BMI (kg/m^2^)	23.8 ± 5.3	25.6 ± 4.3	24.7 ± 4.7	25.9 ± 5.1	23.8 ± 6.8	26.0 ± 5.5	22.9 ± 5.3	25.1 ± 2.9
Ethnicity (number)								
Caucasian	23	18	8	7	5	5	10	6
African American	2	5	1	1	1	0	0	4
Hispanic	0	2	0	1	0	1	0	0
Asian	0	0	0	0	0	0	0	0
Diabetes duration (year)	–	–	–	–	–	–	6.1 ± 5.9	7.6 ± 6.3
HbA_1c_ (% [mmol/mol])	5.8 (4.2) [39.8]	5.6 (2.9) [37.7]	5.4 ± 0.2 [35.5]	5.9 ± 0.3 [40.9]*	5.5 ± 0.1 [36.6]	5.4 ± 0.2 [35.5]	10.7 ± 2.3 [93.4]	11.8 ± 1.8 [105.4]
C-peptide (ng/mL)	1.9 (4.9)	0.5 (3.7)	3.0 ± 1.4	6.7 ± 6.3	11.3 ± 9.9	4.2 ± 4.9	0.1 (0.1)	0.1 (0.3)
Autoantibody positive (number of donors)								
GADA^+^ (IU/ml)	7	12	0	0	6	5	2	6
IA2A^+^ (IU/ml)	5	6	0	0	1	1	4	5
ZnT8A^+^ (IU/ml)	3	5	0	0	0	1	3	4
mIAA^+^ (index)	5	11	0	0	1	1	5	9
Autoantibodies (cum. freq)								
0	13	8	9	9	0	0	3	1
1	6	6	0	0	4	4	2	2
2	4	6	0	0	2	2	3	3
3	2	0	0	0	0	0	2	0
4	0	4	0	0	0	0	0	4
HLA haplotypes (number)								
DR3 allele	8	6	3	2	2	0	2	5
DR4 allele	4	9	2	2	1	4	2	2
DR3/4	6	1	1	1	0	0	5	0

HLA-DR3, HLA-DRB1*03; HLA-DR4, HLA-DRB1*04. Continuous variables that were normally distributed were compared by Student's t-test (shown as mean ± SD). Continuous variables that were non-normally distributed (HbA_1C_, C-peptide) were compared by Mann–Whitney U-test (shown as median (IQR)). Contingency table proportions were analyzed by Fisher's exact test. *p < 0.05 for *GCG*^*hi*^ vs. *GCG*^*lo*^. Cum. freq., cumulative frequency (number).

**Figure 1:**
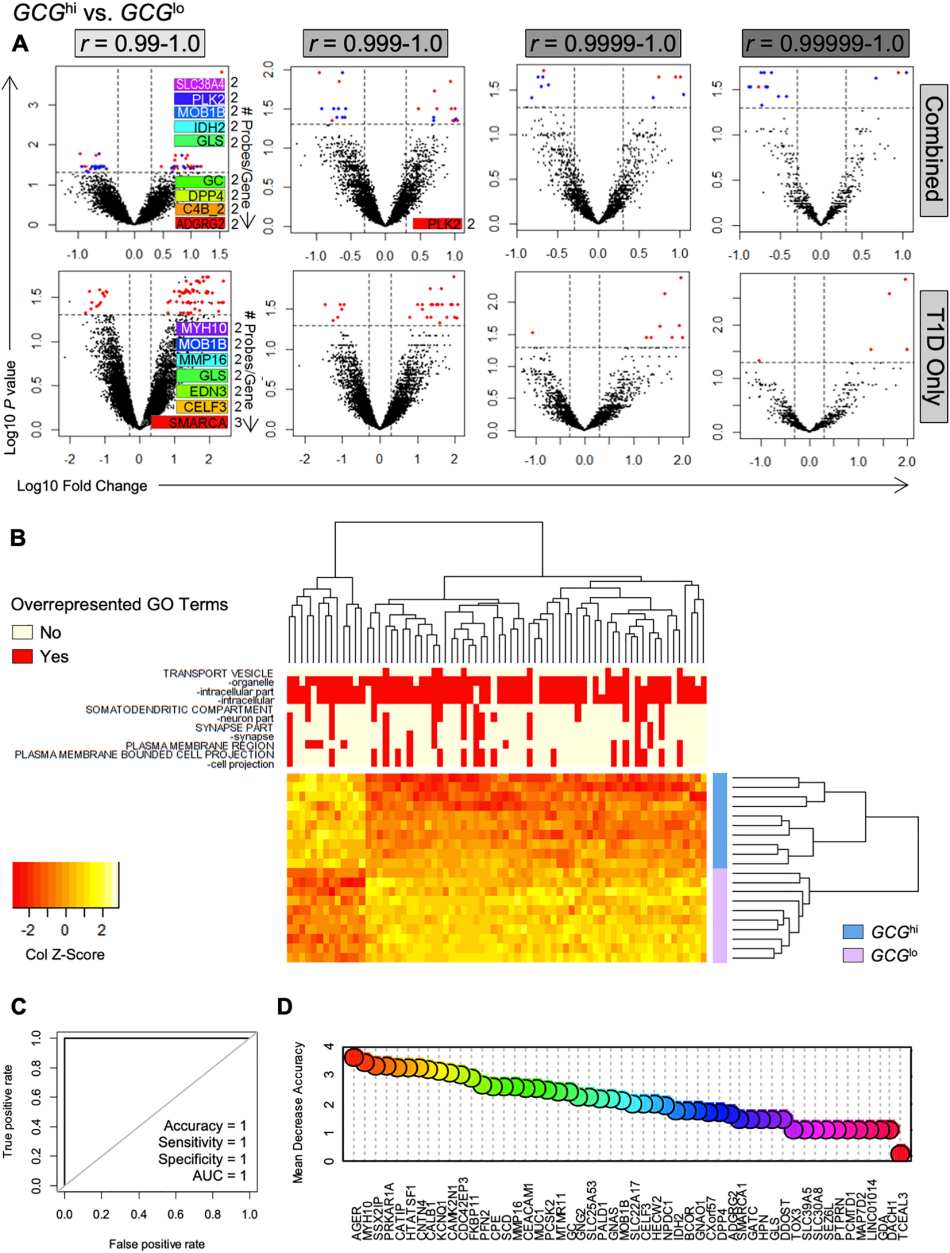
*GCG* expression associates with changes in the expression of *AGER* and its correlated genes in the islets of type 1 diabetes donors. (**A**) Volcano plot of *AGER* and genes correlated with *AGER* at Pearson's *r* = 0.99, 0.999, 0.9999, 0.99999–1.0. Significant differences for control, non-diabetic autoantibody-positive and type 1 diabetes donors combined (*n* = 50; top row), and within the type 1 diabetes cohort alone (*n* = 20; bottom row). Blue dots are significant changes, red dots are significant changes within type 1 diabetes group. See Table S1–S4 for differentially expressed gene lists. No differences were seen within the control or non-diabetic autoantibody-positive groups. Significance was defined by FDR *q* < 0.05, fold-change > 2.0. Where multiple probes detected changes in the expression of one gene (inset rainbow bars, right axes), the probe with the greatest mean expression is shown. (**B**) Unsupervised hierarchically clustered heat maps for differentially expressed genes in type 1 diabetes, which were correlated with *AGER* expression (*r* = 0.99–1.0). Row annotations, *GCG*^hi^ and *GCG*^lo^ subgroups within type 1 diabetes cohort. Column annotations, Gene Ontology enrichment and overrepresentation (Bonferroni adjusted *p* < 0.05). See Figs. S2–S3 for Gene Set Enrichment Analysis (GSEA). (**C,D**) Random forest model trained on 60% (12 of 20) type 1 diabetes donors, then validated on the remaining 40% (8 of 20) type 1 diabetes donors. (**C**) ROC curve for classification into *GCG*^*hi*^ or *GCG*^*lo*^ in validation cohort. (**D**) Variable importance by mean decrease in accuracy.

For *GCG*^hi^ and *GCG*^lo^ subgroups across all donors, volcano plots showed changes in the expression of 48 islet genes (Fig. 1A, top row; FDR *q* < 0.05, fold-change > 2.0), which were significantly correlated with *AGER* expression (*r* = 0.99–1.0). These changes were driven by donors with type 1 diabetes, which when analysed separately there were 13 upregulated and 57 downregulated genes in *GCG*^hi^ donors (as compared to *GCG*^lo^; Fig. 1A, bottom row). Within the type 1 diabetes group, the most significant difference in gene expression between *GCG*^hi^ and *GCG*^lo^ subgroups, was that of the RAGE (*AGER)* gene itself (1.96 log fold-change, FDR *q* = 0.018; Table S1). Control and at-risk autoantibody-positive donors had no significant changes in expression of islet genes between *GCG*^hi^ and *GCG*^lo^ subgroups (FDR *q* > 0.05, fold-change < 2.0, for all; Fig. S1).

To examine the robustness of the selected correlation threshold (*r* = 0.99–1.0), volcano plots were generated using genes that were correlated with *AGER* at increasingly stringent statistical thresholds (Fig. 1A). We found that irrespective of the Pearson's *r* threshold used, the expression of several *AGER* correlated genes remained significantly different between *GCG*^hi^ and *GCG*^lo^ subgroups, across all donors when pooled and this remained significant when only donors with type 1 diabetes were examined (Fig. 1A). These genes are listed in Tables S1-4. Of interest, *AGER* showed the most statistically significant and largest fold-change in gene expression between *GCG*^hi^ and *GCG*^lo^ type 1 diabetes donors, at Pearson's *r* thresholds of 0.999–1.0 (FDR *q* = 0.012), 0.9999–1.0 (FDR *q* = 0.0042) and 0.99999–1.0 (FDR *q* = 0.0014; Tables S1–4). These data suggest that the anchored bioinformatics approach is robust and that expression of *AGER* is significantly associated with *GCG* expression in type 1 diabetes.

### Genes correlated with *AGER* in *GCG*^hi^ type 1 diabetes donor islets are enriched for biological pathways relating to glucagon secretion

Unsupervised hierarchical clustering of *AGER* and its correlated genes that were differentially expressed resulted in clear separation of *GCG*^hi^ and *GCG*^lo^ type 1 diabetes donors (Pearson's *r* = 0.99–1.0; Fig. 1B, row annotations). When these differentially expressed genes were subjected to Gene Ontology enrichment analysis, biological pathways with relevance to glucagon secretion were identified, including pathways entitled "transport vesicles" (GO:0030133; Bonferroni *p* = 4.06 × 10^–2^) and "plasma membrane regions" (GO:0098590; Bonferroni *p* = 1.34 × 10^–3^). Gene Ontology terms within the "transport vesicles" family accounted for most differences in gene expression (Fig. 1B, column annotations). This supports an association between the expression of *AGER* and its correlated genes and that of glucagon secretion in the islets from type 1 diabetes donors.

Gene Set Enrichment Analysis (GSEA) using the Reactome database further identified five significantly enriched biological pathways (nominal *P* ≤ 0.004, FDR *q* < 0.25; Fig. S2), all directly relating to the upregulation of neurotransmitter signaling through glutamate or γ-aminobutyric acid (GABA; Fig. S2).

Using the Gene Ontology database for GSEA, we identified three significantly upregulated cellular components when comparing *GCG*^hi^ and *GCG*^lo^ islets from donors with type 1 diabetes (nominal *P* ≤ 8 × 10^–3^, FDR *q* < 0.25; Fig. S3). These were "heterotrimeric G protein complex", which has a known role in glucagon release^24^, "protein transporting two sector ATPase complex", which generates ATP that is critical for the secretion of glucagon^25^, and "ciliary tip", which is also an important aspect of the glucagon secretion pathway^26^. Altogether, these data further support a role for *AGER* and its correlated genes in modulating biological pathways and cellular components with relevance to the secretion of glucagon in type 1 diabetes.

### Random forest machine learning identifies islet *AGER* as an important predictor for *GCG* expression in type 1 diabetes

We utilized random forest ensemble machine learning to generate a model to explain the expression of *GCG* using *AGER* and its correlated genes in islets from donors with type 1 diabetes. To this end, a random forest model trained on 60% (12 of 20) of the type 1 diabetes cohort (out of bag error rate, 8.33%), achieved 100% accuracy in classifying donors into *GCG*^hi^ and *GCG*^lo^ subgroups in the remaining 40% (8 of 20) type 1 diabetes donors, which was the validation cohort (Fig. 1C). The most important variable determined by mean decrease in accuracy was *AGER* (Fig. 1D) i.e., the loss in machine learning accuracy in predicting the expression of *GCG* when a single gene was excluded. These findings support the notion that the expression of *AGER* and *GCG* are associated in islets from donors with type 1 diabetes.

### Generalized linear model using RAGE ligands and pathways predicts *GCG* expression in type 1 diabetes

To perform the converse analyses, we used generalized linear modelling (GLM) to see if islet *GCG* expression could be predicted in donors with type 1 diabetes using RAGE ligands and downstream signaling pathways for RAGE, instead of using the expression of *AGER* itself. Here, the starting model was *GCG* = *HMGB1* + *AKR1B1* + *FN3K* + *RELA* + *JAK1* + *STAT3* + *MAPK1*. It included the RAGE ligand *HMGB1*, as well as *AKR1B1*, which is the rate-limiting enzyme in the polyol pathway that generates the RAGE ligand AGEs, and *FN3K*, which is a major AGE clearance enzyme. The RAGE signaling pathways included were *RELA, JAK1*/*STAT3* and *MAPK1.*

**Figure 2:**
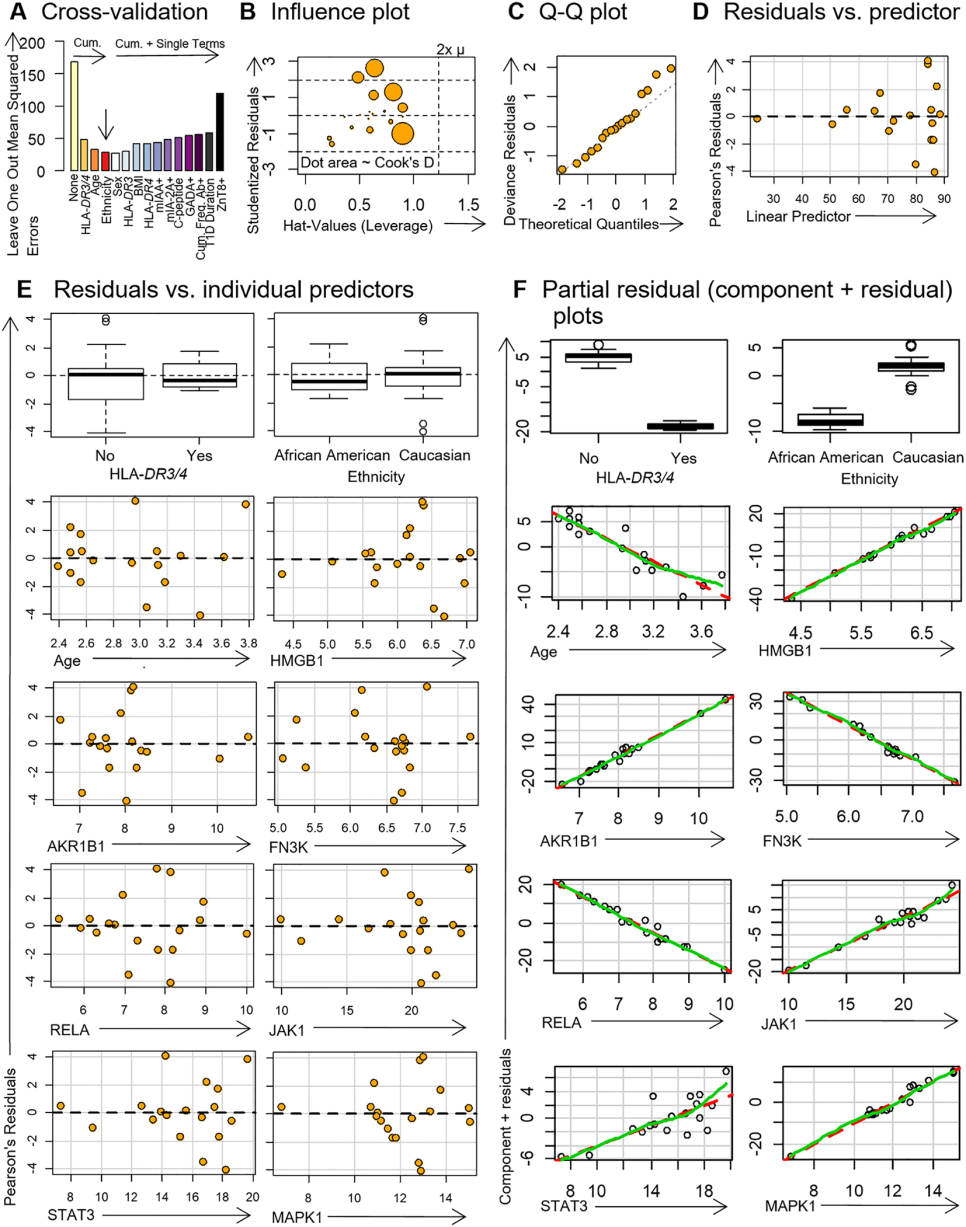
Biostatistical diagnostic plots for generalized linear model (GLM; Gaussian identity-linked) of *GCG* expression in islets from donors with type 1 diabetes. (**A**) Donor characteristics were added one-by-one to the starting model (*GCG* = *HMGB1* + *AKR1B1* + *FN3K* + *RELA* + *JAK1* + *STAT3* + *MAPK1*). Donor characteristics were retained in the model if it resulted in the largest reduction in leave-one-out cross-validation mean squared error (LOOCV MSE). This was repeated for the cumulative addition of donor characteristics. When the LOOCV MSE only increased, the model was considered parsimonious (vertical arrow) so no further donor characteristics were included. (**B**) Influence plot for outlier identification. Bubble area is proportional to Cook's D statistic (donors #6196 and #6084 were outliers and excluded because Cook's D ≥ 1). (**C**) Quantile–quantile (Q–Q) plot showed the residuals were reasonably normally distributed. (**D**) Residuals vs. linear predictor plot showed uniform heteroskedasticity (robust errors were used). (**E**) Residuals vs. individual predictors showed uniform heteroskedasticity. (**F**) Partial residual (component + residual) plots showed linearity between individual predictors and *GCG* expression (green, Loess line; red, component line). Final model was *GCG* = HLA-*DR3/4* + Age + Ethnicity + *HMGB1* + *AKR1B1* + *FN3K* + *RELA* + *JAK1* + *STAT3* + *MAPK1*. Donor characteristics were log-transformed, gene expression values were square-root transformed. See Table 3 for GLM.

To account for model confounders, donor characteristics were introduced into the starting model one-by-one. Using leave one out cross validation (LOOCV), we determined that the inclusion of HLA-*DR3/4* status, age and ethnicity decreased the LOOCV mean squared error (MSE; Fig. 2A), thereby improving the model fit^27^. By contrast, the remaining donor characteristics contributed to model overfitting, as determined by an increased LOOCV MSE (Fig. 2A). The final parsimonious model that did not unnecessarily overfit was *GCG* = HLA-*DR3/4* + Age + Ethnicity + *HMGB1* + *AKR1B1* + *FN3K* + *RELA* + *JAK1* + *STAT3* + *MAPK1.*

To avoid inaccurate regression estimates due to outlier bias (Cook's D > 1.0; Fig. 2B), donors #6196 and #6084 were excluded from the model. Residuals were normally distributed (Fig. 2C) but had uniform heteroskedasticity (i.e., cone-like distributions in Fig. 2D, E) so we used robust standard errors. Partial residual plots showed linearity between predictors and *GCG*, which supports the use of predictors with no exponents or powers (Fig. 2F).

After correction for *HLA-DR3/4* haplotype status, ethnicity and age, the final model found that in the type 1 diabetes cohort, increased *GCG* expression could be predicted by increased expression of *HMGB1* and *AKR1B1*, as well as by reduced expression of the AGE clearance enzyme, *FN3K* (Table 3; FDR *q* < 0.0001 for all)*.* Further, *RELA* was inversely associated with *GCG* expression, whereas *JAK1* and *MAPK1* were both positively associated with *GCG* (Table 3; FDR *q* < 0.0001 for all). The expression of *STAT3* did not predict *GCG* expression (Table 3; FDR *q* = 0.078). Taken together, these results from microarray bioinformatics are consistent with an association between the expression of *AGER*, its ligands, signaling pathways and correlated genes and the expression of *GCG* in type 1 diabetes.

**Table 3:** Generalized linear model (GLM) for islet expression of *GCG* in type 1 diabetes.

Independent variables	Regression coefficient, β		*p* value	FDR *p* value
	Estimate	Robust standard error		
HLA-*DR3/4*				
No	Reference	–	–	–
Yes	−23.7044	1.0174398	4.63125 × 10^–120^	2.31562 × 10^–119^ ****
Ethnicity				
Afr. American	Reference	–	–	–
Caucasian	9.5167	2.4162714	8.19572 × 10^–05^	9.70665 × 10^–09^ ****
Age	−11.3032	1.9577730	7.76532 × 10^–09^	9.10636 × 10^–05^ ****
*HMGB1*	21.5527	0.9708416	3.43683 × 10^–109^	1.14561 × 10^–108^ ****
*AKR1B1*	16.4209	1.0387994	2.75746 × 10^–56^	6.89365 × 10^–56^ ****
*FN3K*	−25.3323	0.9121661	9.56227 × 10^–170^	9.56227 × 10^–169^ ****
*RELA*	−9.5669	0.7792199	1.19517 × 10^–34^	2.39035 × 10^–34^ ****
*JAK1*	2.1058	0.3607738	5.31949 × 10^–09^	7.59927 × 10^–09^ ****
*STAT3*	0.7556	0.4300628	7.89136 × 10^–02^	7.89136 × 10^–02^
*MAPK1*	5.0048	0.4434898	1.55389 × 10^–29^	2.58982 × 10^–29^ ****

Donor islets were laser captured from pancreata by nPOD from organ donors without diabetes (control, *n* = 18), autoantibody-positive donors without diabetes (*n* = 12) and donors with type 1 diabetes (*n* = 20). Gene expression microarrays and generalized linear modelling was performed to predict islet glucagon expression. The final model (*GCG* = HLA-*DR3/4* + Age + Ethnicity + *HMGB1* + *AKR1B1* + *FN3K* + *RELA* + *JAK1* + *STAT3* + *MAPK1*) was statistically significant, when compared to the null model (*p* < 2.2 × 10^–16^ by χ^2^-test; Akaike's Information Criterion, AIC = 100.8). The RAGE ligands S100A8/9/B were not added to the final model because they did not improve goodness-of-fit (AIC = 102.1). Robust standard errors were used to account for the uniform heteroskedasticity of residuals. Donor characteristics were log-transformed, gene expression values were square-root transformed. See Fig. 2 for model validation plots.

Null deviances (degrees of freedom, df), 4865 (17). Final model's residual deviances (df), 75.1 (7).

### α cells from type 1 diabetes donor islets have increased glucagon, RAGE, CML and cytosolic-to-nuclear HMGB1

**Figure 3:**
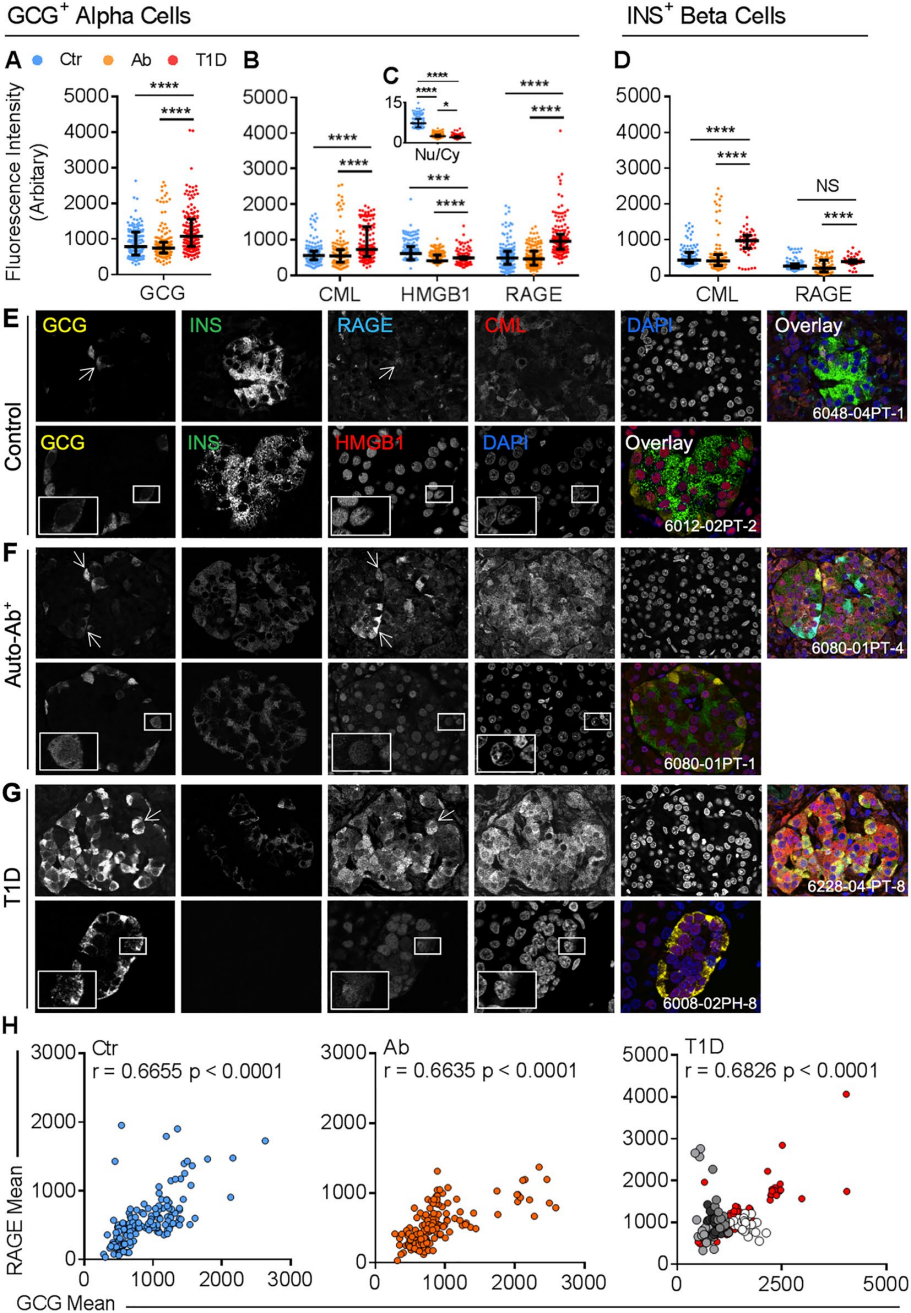
α cell expression of glucagon and RAGE is increased in type 1 diabetes but inversely associated in adolescents with type 1 diabetes (T1D-adolescents). (**A–G**) α cell expression of (**A**) glucagon, (**B**) CML, HMGB1, RAGE and the (**C**) HMGB1 nuclear/cytoplasmic ratio (inset) were analyzed by quantitative confocal microscopy. Control (Ctr; ages 14–68, BMI 21–30), non-diabetic autoantibody-positive (Auto-Ab^+^ or Ab; ages 4.4–69, BMI 14.8–34.3) or type 1 diabetes donors (ages 5–37.2, BMI 17.4–30.9) were compared by one-way ANOVA (*n* = 15–20 islets/donor, *n* = 8 donors/group). (**D**) β cell expression of CML and RAGE were analyzed by quantitative confocal microscopy. (**E–G**) Representative photomicrographs of (**E**) control (Ctr), (**F**) non-diabetic autoantibody-positive (Auto-Ab^+^ or Ab), and (**G**) type 1 diabetes donors. Panels (**F,G**) are the same markers as shown in panel (**E**). (**H**) Correlation analyses between glucagon and RAGE expression. Pearson’s *r* for the control (Ctr), non-diabetic autoantibody-positive (Auto-Ab^+^ or Ab) and type 1 diabetes cohorts are shown. Pearson’s *r* for individual donors are in Fig. S4. For individual donors, statistically significant negative Pearson’s *r* (hereby, referred to as T1D-adolescents) are shown as enlarged monochromatic dots (type 1 diabetes donors #6228, 6046, 6196, 6195; white to dark grey). Data in (**A–D**) were compared by Kruskal–Wallis and Dunn’s post-hoc test, and shown as median and interquartile range. Data in (**H**) were analyzed by Pearson’s correlation. *p < 0.05; **p < 0.01; ***p < 0.001; ****p < 0.0001; NS, not significant. Images captured with a 60 × oil-immersion lens (NA 1.35); numerical identifiers inset in the images are de-identified participant codes from the nPOD repository.

To confirm our bioinformatics findings, we performed quantitative immunofluorescence (IF) of human donor pancreata. Here, we found that GCG expression was increased in the islets of donors with type 1 diabetes, as compared with control and at-risk autoantibody-positive donors (Fig. 3A, shown in Fig. 3E–G). In the α cells, the RAGE ligand, CML which is an advanced glycation end product (AGE) was also increased in islets from donors with type 1 diabetes (vs. remaining donor groups; Fig. 3B, shown in Fig. 3E–G), whereas HMGB1 staining intensity was reduced in both autoantibody-positive and type 1 diabetes donors (vs. control; Fig. 3B, shown in Fig. 3E–G). Further, HMGB1 had translocated from the nucleus into the cytoplasm in both the autoantibody-positive and type 1 diabetes donor islets (Fig. 3C, shown in Fig. 3E–G) when compared with control donors. There were also concomitant increases in α cell RAGE expression in type 1 diabetes (Fig. 3B, shown in Fig. 3E–G). Both RAGE and CML staining in the β cells within islets from donors with type 1 diabetes were also increased (Fig. 3D), but here, the changes in RAGE expression were far less pronounced than seen in the α cells (Fig. 3B).

### α cell RAGE and glucagon are inversely correlated in a significant proportion of adolescents with type 1 diabetes

We also examined the relationship between GCG and RAGE in the α cells. We found that RAGE positively correlated with GCG expression within the control, autoantibody-positive and type 1 diabetes groups (Fig. 3H; *p* < 0.0001 for all). Interestingly, we only reported this positive association in the type 1 diabetes donors by microarray, but identified a strong positive correlation between islet RAGE and glucagon protein levels in all donor groups by confocal microscopy.

**Figure 4:**
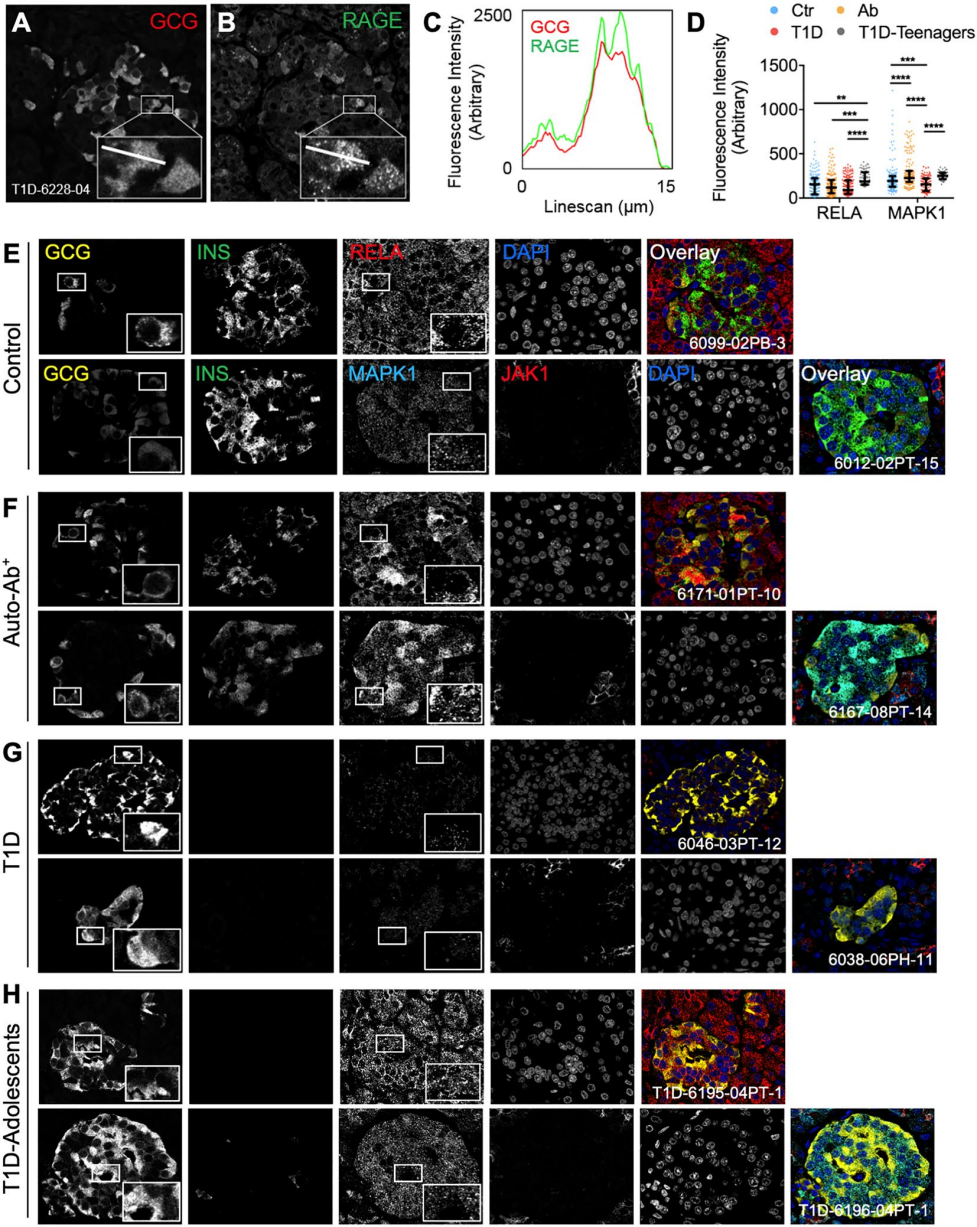
α cell expression of RELA and MAPK1 are reduced in type 1 diabetes but increased in T1D-adolescents donors. (**A–C**) Representative line scan analysis of (**A**) glucagon and (**B**) RAGE expression in type 1 diabetes. (**C**) Line scan histogram. (**D**) α cell expression of RELA and MAPK1 were analyzed by quantitative confocal microscopy. Control (Ctr; ages 14–68, BMI 21–30), non-diabetic autoantibody-positive (Auto-Ab^+^ or Ab; ages 4.4–69, BMI 14.8–34.3), type 1 diabetes donors (ages 5–37.2, BMI 22–30.9) or T1D-adolescents (ages 13–26.5, BMI 17.4–26.6) were compared by one-way ANOVA (*n* = 15–20 islets/donor, *n* = 8 donors/group). (**E–H**) Representative photomicrographs of (**E**) control (Ctr), (**F**) non-diabetic autoantibody-positive (Auto-Ab^+^ or Ab), (**G**) type 1 diabetes and (**H**) T1D-adolescents. Panels (**F–H**) are the same markers as shown in panel (**E**). Data in (**D**) were compared by Kruskal–Wallis and Dunn’s post-hoc test, and shown as median and interquartile range. *p < 0.05; **p < 0.01; ***p < 0.001; ****p < 0.0001; *NS* not significant. Images captured with a 60× oil-immersion lens (NA 1.35); numerical identifiers inset in the images are de-identified participant codes from the nPOD repository.

To further examine the relationship between GCG and RAGE, we measured fluorescent intensities of each unique islet within individual donor tissue sections. To this end, we observed a phenomenon where half of all type 1 diabetes donors showed a significant negative association between RAGE and GCG fluorescent intensities in single islets (4 of 8 type 1 diabetes donors, Fig. 3H), whereas this was not seen in the absence of diabetes. Interestingly, the propensity of islets to have a negative correlation between RAGE and GCG expression was only present in adolescents from the type 1 diabetes cohort (donors #6228, #6046 and #6195, who were aged 13, 18 and 19 years respectively) and donor #6228—while not an adolescent—had recent history of rapid weight loss and familial hypoglycemia. To establish cellular colocalization of GCG and RAGE, we performed line scan analysis where we found that GCG and RAGE were co-expressed in comparable domains of the α cells in islets from donors with type 1 diabetes (Fig. 4A–C).

### Type 1 diabetes adolescent donors with an inverse relationship between α cell RAGE and glucagon have increased α cell expression of RELA and MAPK1

To further examine the association between RAGE and glucagon expression, we used quantitative immunofluorescence to examine downstream RAGE pathways that were significant predictors for GCG expression in the microarray GLM. We found that MAPK1, but not RELA, was significantly reduced in the α cells of donors with type 1 diabetes (vs. control and autoantibody-positive donors; Fig. 4D, shown in Fig. 4E–G), whereas JAK1 was expressed at insignificant levels within the islets in all groups (Fig. 4E–G).

Interestingly, we found that in type 1 diabetes donors with an inverse relationship between RAGE and GCG (hereby, T1D-adolescents), there was increased α cell RELA (vs. remaining type 1 diabetes donors, Fig. 4D, shown in Fig. 4G–H). We observed a similar increase in MAPK1 expression in the α cells of T1D-adolescents, as compared with the remaining type 1 diabetes donors (Fig. 4D, shown in Fig. 4G–H). Taken together, these data show an association between RAGE and GCG expression in the α cells in type 1 diabetes, in which there was an inverse correlation in a donor with documented hypoglycemia as well as in adolescents who are clinically at higher risk of this potentially lethal complication.

## Discussion

In type 1 diabetes, the α cells have impaired glucagon secretion and an altered gene expression profile (11). However, the mechanisms contributing to α cell dysfunction in the development and progression of type 1 diabetes remain poorly characterized. Here, we observed the following in donor pancreata from those with type 1 diabetes; (1) islet *GCG* expression is associated with increases in *AGER* and changes in *AGER* correlated genes, (2) genes correlated with *AGER* in *GCG*^*hi*^ donor islets are enriched for biological pathways relating to glucagon secretion, (3) islet *AGER* is a predictor for *GCG* expression, (4) a generalized linear model including RAGE ligands and signaling pathways explains *GCG* expression, (5) α cells have increased glucagon, RAGE, CML and cytosolic-to-nuclear HMGB1, (6) α cell RAGE and glucagon are inversely correlated in adolescents and in a donor with documented hypoglycemia, and (7) donors with an inverse relationship between α cell RAGE and glucagon have increased islet *RELA* and *MAPK1*.

The relationship between glucagon and RAGE was of interest in the islets of adolescent donors with type 1 diabetes as they were more recently diagnosed and had shorter disease duration. Here, we identified a negative association between islet RAGE and glucagon expression in the adolescents within the type 1 diabetes cohort, which was not seen in the absence of diabetes. This has not been previously described. In various models of diabetes as well as in human islets, changes in islet RAGE expression have been reported. Indeed, RAGE has been identified in the islets of people with and without type 2 diabetes^28^. We had also shown that early in type 1 diabetes development in mice, there was a decrease in islet RAGE expression on α cells as well lower glycated hemoglobin concentrations^17^. These studies suggest that there may be an association between hypoglycemia, glucagon and RAGE expression in the islets in type 1 diabetes that warrants further exploration in future studies.

In donors with type 1 diabetes, we found an increase in α cell expression of RAGE by both microarray and confocal microscopy. Conversely, RAGE protein expression, but not RNA levels, were increased in the control and autoantibody-positive donor islets based on glucagon stratification. This could reflect the inherent differences in RNA and protein regulation, varying sensitivities of the research techniques used or physiologically relevant reasons such the upregulation of cleavage processes for islet preproglucagon^29^. While there are limited studies examining the effects of the RELA/NF-κB and MAPK cascades on glucagon expression, it is highly likely that these signaling pathways play a role in the differences described above. Finally, it could be interesting in the future to explore if and how any residual β cell function and exogeneous insulin use affects the findings described here, as there is accumulating evidence that is suggestive of discrete endotypes in type 1 diabetes^30^.

Increases in tissue RAGE expression are often seen after the binding of RAGE ligands such as CML and HMGB1^31^. In the present study, the RAGE ligand CML was also increased in islets from donors with type 1 diabetes, which is consistent with previous data in rodent models^32,33^. Conversely, HMGB1 expression was reduced, with its subcellular localization shifted from the nucleus into the cytoplasm. However, this is consistent with the release of nuclear HMGB1 into the cytoplasm, which is an activation pathway that precedes its binding to RAGE, providing further support that RAGE ligand binding and signaling is increased in these donor individuals with type 1 diabetes^34^.

**Figure 5:**
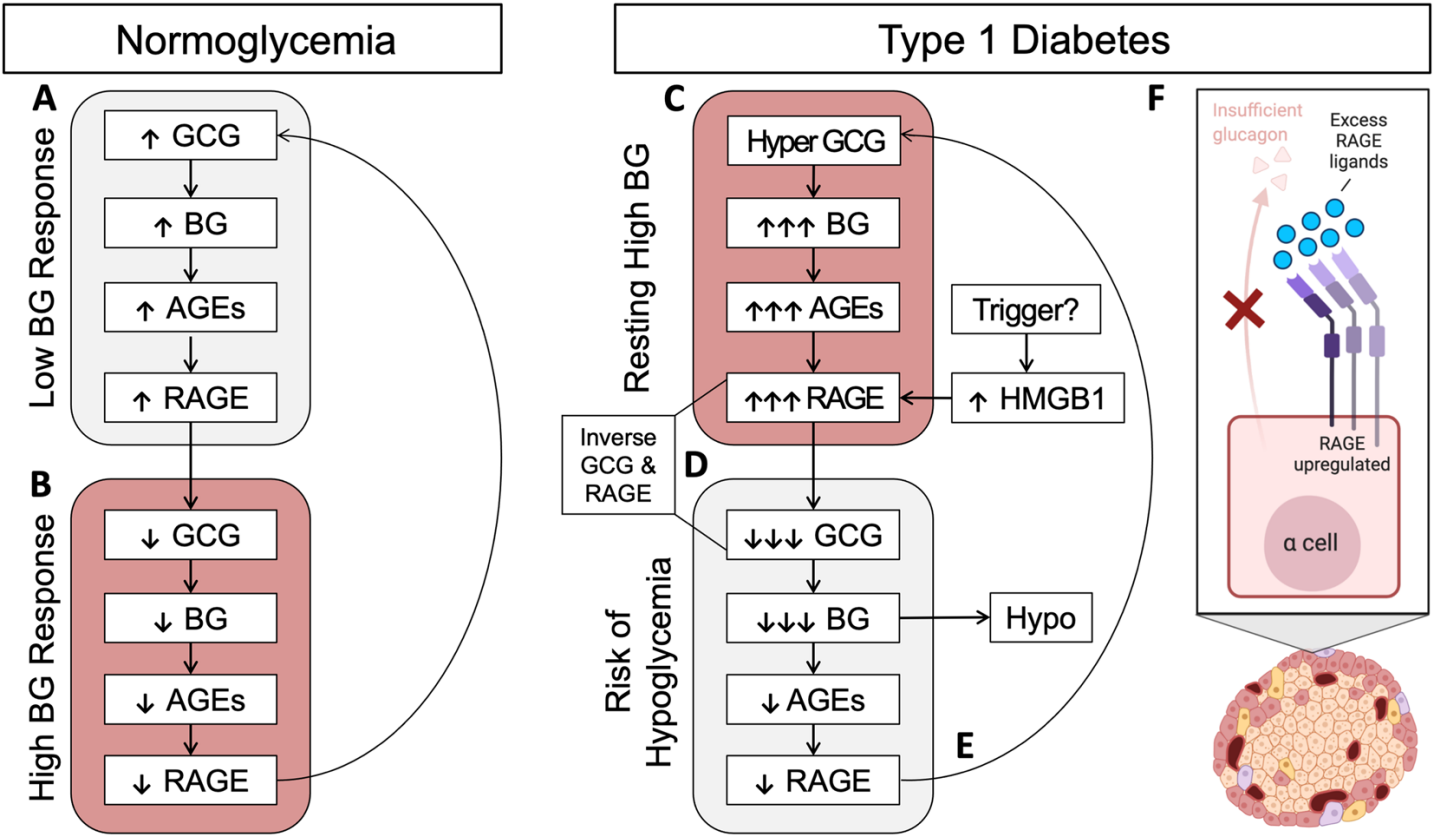
Schematic of RAGE as an α cell sensor that regulates the expression of glucagon. (**A**) In non-diabetic conditions, glucagon (GCG) expression leads to increased blood glucose (BG) levels, which generates AGEs, thereby binding and upregulating RAGE. (**B**) Then, RAGE signaling suppresses further glucagon expression, decreasing blood glucose levels, AGEs and RAGE expression. (**C**) In type 1 diabetes, resting hyperglucagonemia leads to hyperglycemia, excessive AGEs, excessive RAGE binding and its upregulation. (**D**) Then, excessive RAGE signaling leads to significant suppression of glucagon secretion, creating a high-risk for hypoglycemia characterizing the type 1 diabetes adolescents in this study i.e., an inverse correlation in GCG and RAGE expression. Significant decreases in blood glucose occur, which is followed by decreases in AGEs and RAGE. (**E**) Eventually, a compensatory period is observed that re-establishes hyperglucagonemia and high blood glucose levels. (**F**) Illustration of proposed RAGE and glucagon relationship in type 1 diabetes (created with Biorender.com).

Overall, these data illustrate an association between glucagon and RAGE in the α cells, with a particularly novel relationship in adolescents with type 1 diabetes. The timing for this relationship is interesting as it suggests a role in the loss of glucagon counterregulation, which has been observed as soon as 1-month after the early diagnosis of type 1 diabetes^29^. However, these findings warrant further investigation in future studies. The RAGE ligands, AGEs, are constantly found in the circulation including HbA1c, fructosamine albumin and CML, all of which are well known to be elevated in diabetes. While many of these markers are clinically used to assess blood glucose control, the reason for their presence at lower levels in the circulation under physiological conditions remains to be fully elucidated. Elevations in circulating CML also confer risk for type 1 diabetes development in school aged children, above the level of risk found with autoantibodies^35^. Thus, it is tempting to speculate that under physiological conditions, RAGE signaling could functionally impact glucagon expression in the α cells. One potential hypothesis is that increases in RAGE signaling after ligand binding by circulating AGEs under conditions where blood glucose is elevated^31^, signals to decrease blood glucose concentrations by the suppression of glucagon expression (Fig. 5A, B). This should be the focus of future studies in this area.

By contrast, in diabetes where the α cells are dysfunctional, excessive RAGE ligands and islet RAGE upregulation leads to chronic activation of RAGE and the pathological suppression of glucagon expression (Fig. 5C, D). This period of elevated RAGE activation and excessive glucagon suppression may reflect the inverse relationship between glucagon and RAGE seen in the adolescent donors with type 1 diabetes. We propose that this may contribute to a dysfunctional glucagon response to hypoglycemia and compensatory hyperglucagonemia under basal conditions (Fig. 5E; Fig. 5F shown as an illustration). Ultimately, this postulate requires further validation and functional studies in the future to confirm.

This study identified a novel association between glucagon and RAGE in human islets, including in type 1 diabetes. In RNA microarray, RAGE (*AGER*) gene expression was the most important predictor of islet glucagon expression in type 1 diabetes. Islet RAGE, its ligands and signaling molecules were also significantly associated with glucagon levels in microarray and immunofluorescence analyses. A negative correlation between glucagon and RAGE expression in the islets was found in 50% of type 1 diabetes donors, including three adolescents and a donor with a clinical history of hypoglycemia, alluding to a possible link between islet RAGE expression and the risk of a hypoglycemic event. Finally, we presented a potential model for RAGE as a glucose sensor that is dysregulated in type 1 diabetes. Further studies are warranted to confirm if the association between RAGE and the modulation of glucagon expression seen here is functionally reproducible in human islets.

### Supplementary Information

Supplementary Information.

## Data Availability

The datasets generated during and/or analyzed during the current study are available from the corresponding author on reasonable request.
