# Supplementary material for: Alpha cell receptor for advanced glycation end products associate with glucagon expression in type 1 diabetes
Source: Sci Rep. 2023 Aug 9;13:12948. doi: 10.1038/s41598-023-39243-x (PMC10412557; doi:10.1038/s41598-023-39243-x)
Supplement: Supplementary file 1 — Supplementary Information. [file 41598_2023_39243_MOESM1_ESM.pdf]

Online Supplemental Material for Leung et al "Alpha Cell Receptor for Advanced Glycation End Products Associate with Glucagon Expression In Type 1 Diabetes"

Supplemental Information Titles and Legends

Supplemental Methods. Generalized linear model (GLM) bioinformatic methods.

Table S1. Changes in the expression of *AGER* correlated genes for GCG^{hi} vs. GCG^{lo} islets (Pearson's *r* = 0.99-1.0; 20,937 probe sets), related to Figure 1A. Changes are shown as log fold change, P values are FDR corrected. See Figure 1A for volcano plot visualization.

Table S2. Changes in the expression of *AGER* correlated genes for GCG^{hi} vs. GCG^{lo} islets (Pearson's *r* = 0.999-1.0; 9228 probe sets), related to Figure 1A. Changes are shown as log fold change, P values are FDR corrected. See Figure 1A for volcano plot visualization.

Table S3. Changes in the expression of *AGER* correlated genes for GCG^{hi} vs. GCG^{lo} islets (Pearson's *r* = 0.9999-1.0; 3055 probe sets), related to Figure 1A. Changes are shown as log fold change, P values are FDR corrected. See Figure 1A for volcano plot visualization.

Table S4. Changes in the expression of *AGER* correlated genes for GCG^{hi} vs. GCG^{lo} islets (Pearson's *r* = 0.99999-1.0; 1064 probe sets), related to Figure 1A. Changes are shown as log fold change, P values are FDR corrected. See Figure 1A for volcano plot visualization.

Figure S1. *GCG* expression does not associate with changes in the expression of *AGER* and its correlated genes in the islets of control and non-diabetic autoantibody-positive donors, related to Figure 1A.
Control (n = 18) and non-diabetic autoantibody-positive (n = 12) donors. Significance was defined by FDR $q < 0.05$, fold-change > 2.0.

Figure S2. Gene Set Enrichment Analysis (GSEA) using Reactome Pathway Database for *AGER* and its correlated genes in GCG^{hi} vs. GCG^{lo} islets, related to Figure 1B.
(A-E) GSEA enrichment score curves. NES = normalized enrichment score, Nom p = nominal p value, FDR q = false discovery rate q values. *AGER* and its correlated genes were selected on a Pearson's *r* threshold of 0.99-1.0 (20,937 probe sets).

Figure S3. GSEA using Gene Ontology Cellular Components for *AGER* and its correlated genes in GCG^{hi} vs. GCG^{lo} islets, related to Figure 1B.
(A-E) GSEA enrichment score curves. NES = normalized enrichment score, Nom p = nominal p value, FDR q = false discovery rate q values. *AGER* and its correlated genes were selected on a Pearson's *r* threshold of 0.99-1.0 (20,937 probe sets).

Figure S4. Correlation analyses of glucagon and RAGE expression in the α cells for individual donors by quantitative confocal microscopy, related to Figure 3H.

(A-D) Pearson's r for the control (Ctr), non-diabetic autoantibody-positive (Auto-Ab^+ or Ab) and type 1 diabetes cohorts are shown. P values are indicated in the figure; NS, not significant. Pearson's r for the Ctr, Ab and type 1 diabetes groups overall are in Figure 3H.

Supplemental Methods. Generalized linear model (GLM) bioinformatic methods.

Generalized linear modelling (GLM) was used to test the hypothesis that glucagon expression can be predicted by the expression of RAGE ligands and signalling molecules. The starting model had the following variables added in forward stepwise selection (*GCG* = *HMGB1* + *ARK1B1* + *FN3K* + *RELA* + *JAK1* + *STAT3* + *MAPK1*) as each variable improved the Akaike Information Criterion (AIC) i.e., the model's goodness-of-fit [1]. The RAGE ligands S100A8/9/B were excluded as they did not improve AIC. Statistical significance for the GLM was confirmed by χ^2-test, as compared to the null model.

Clinical variables in Tables 1-2 were considered possible confounders, so these were assessed for inclusion by leave-one-out cross validation (LOOCV). In LOOCV, if variable inclusion resulted in the largest average reduction in the mean square error (MSE) for predicting glucagon expression in the one subject that was 'left out' of the training dataset (repeated forty times i.e., a unique subject 'left out' in each repetition), it was considered a significant confounder and added to the model [2]. This was repeated until it increased the average MSE i.e., the addition of further clinical data in Tables 1-2 led to model overfitting, worsening its ability to accurately predict a subject's glucagon expression.

Diagnostic plots were used to assess GLM performance in the *car* package in R [3]. These included an influence plot for the identification of outliers by Cook's D, quantile-quantile (Q-Q) plot to visualize the normal distribution of residuals, residuals vs. predictors plot to visualize heteroskedasticity (i.e., *robust,* instead of *standard*, errors to be used [4]) and partial residual plots to visualize linearity between predictors and *GCG* expression (i.e., higher order powers not required in the GLM).

References

1. Zar, J. H. Biostatistical analysis 5th edition (Prentice-Hall/Pearson, 2010).
2. Lever, J., Krzywinski, M., Altman, N. Model selection and overfitting. *Nature Methods* **13** (2016).
3. Fox J., Weisberg S. An R companion to applied regression 2nd Edition (Thousand Oaks CA: Sage, 2011).
4. Zeileis A. Econometric computing with HC and HAC covariance matrix estimators. *Journal of Statistical Software* **11** (2004).

Table S1. Changes in the expression of *AGER* correlated genes for GCG^{hi} vs. GCG^{lo} islets (Pearson's *r* = 0.99-1.0; 20,937 probe sets). Changes are shown as log fold change, P values are FDR corrected. See Figure 1A for volcano plot visualization.

Gene Symbol	Change	P value
**All donors (48 genes)**		
**Downregulated**		
FKBP11	-0.9516	0.0171
TTLL12	-0.6108	0.0171
HPN	-0.6668	0.0190
PLTP	-0.9200	0.0349
DDOST	-0.8760	0.0349
EIF3J	-0.7432	0.0349
LMAN2	-0.7004	0.0349
MYH9	-0.6788	0.0349
C4B_2	-0.6784	0.0349
CLDN3	-0.6148	0.0349
ATF4	-0.5876	0.0349
FLAD1	-0.5736	0.0349
FBXL18	-0.5652	0.0349
AGTRAP	-0.5316	0.0349
SPCS3	-0.6732	0.0385
CDC42EP1	-0.6240	0.0396
IDH2	-0.7712	0.0400
JOSD2	-0.6904	0.0402
FAM129B	-0.4900	0.0416
LGALS4	-0.8368	0.0479
RPLP0	-0.8176	0.0498
**Upregulated**		
GCG	1.5444	0.0002
SMARCA1	0.7268	0.0190
DLG4	0.8448	0.0190
ANKS1B	0.9444	0.0190
MYH10	0.9176	0.0224
FXYD5	0.8748	0.0349
MOB1B	1.0460	0.0349
AGER	0.9520	0.0349
PHC3	0.4732	0.0349
PRKAR1A	0.4764	0.0349
CEACAM1	0.7428	0.0349
CACNA2D2	0.7636	0.0349
FAP	1.1036	0.0349
GC	1.1908	0.0349
NECAB2	0.7156	0.0349
PTPN13	0.7000	0.0349
SLC38A4	0.9928	0.0372
RTN1	1.0264	0.0372
KCNMA1	0.8912	0.0385
PLCB4	1.0060	0.0385
EPB41L3	0.6632	0.0389
PLK2	1.0504	0.0402
PCSK2	0.7060	0.0416
DPP4	1.1672	0.0416
ADGRG2	1.0076	0.0416
GLS	1.0140	0.0417
ASTN1	0.6732	0.0498

Gene Symbol	Change	P value
**Type 1 diabetes donors (70 genes)**		
**Downregulated**		
CALB1	-1.1550	0.0259
CATIP	-0.9700	0.0272
KCNQ1	-1.4460	0.0272
PCMTD1	-0.9400	0.0278
HPN	-1.0590	0.0287
SLC39A5	-1.0430	0.0288
SCRIB	-1.0130	0.0309
DDOST	-1.3770	0.0365
IDH2	-1.1190	0.0365
PALD1	-1.1750	0.0386
FKBP11	-1.2390	0.0406
AMBP	-1.3860	0.0434
MUC1	-1.5460	0.0478
**Upregulated**		
AGER	1.9690	0.0187
STX1A	1.8150	0.0187
SLC22A17	1.6310	0.0209
HTATSF1	1.3420	0.0209
GCG	2.4150	0.0209
NPDC1	1.3280	0.0209
GNG2	1.6870	0.0209
SSX2IP	1.8370	0.0209
CDC42EP3	1.7190	0.0215

UTP14C	1.4720	0.0224
DACH1	1.5140	0.0259
MYO3A	1.3380	0.0259
BCOR	0.9990	0.0272
GATC	1.3540	0.0272
DPP4	1.1410	0.0272
GNAS	1.3020	0.0272
MTMR11	1.1460	0.0272
CAMK2N1	1.6560	0.0272
CELF3	1.9780	0.0272
MMP16	1.5450	0.0272
HECW2	1.4050	0.0275
ADGRG2	1.8950	0.0287
SMARCA1	2.0810	0.0287
IDS	1.2270	0.0287
MYH10	1.5640	0.0287
APLP1	1.4560	0.0287
GNAO1	1.2750	0.0287
PLK2	1.1430	0.0309
TCEAL3	0.8730	0.0350
PFN2	1.7890	0.0359
GDA	1.0680	0.0359
TOX3	1.4110	0.0359
MOB1B	1.9490	0.0359
PRKAR1A	0.8030	0.0365
CEACAM1	1.3600	0.0365
GC	2.4120	0.0365
PCSK2	2.3540	0.0365
GLS	1.9340	0.0365
SEZ6L	1.2540	0.0365
SHFM1	1.3920	0.0365
CXorf57	1.7900	0.0365
PTPRN	2.2670	0.0365
ABCC9	1.5860	0.0368
CA8	1.4500	0.0368
SCD	2.0650	0.0368
ANKS1B	1.5930	0.0434
EDN3	1.9980	0.0452
CPE	2.2690	0.0460
ZNF335	1.3730	0.0472
LINC01014	1.2650	0.0478
SLC30A8	1.2630	0.0478
CNTN4	1.4560	0.0478
VPS13C	1.1760	0.0478
SLC25A53	1.4800	0.0478
RAB11A	0.8230	0.0478
ST18	0.8130	0.0478
MAP7D2	1.3960	0.0498

Gene Symbol	Change	P value
**All donors and type 1 diabetes donors only (18 genes in common)**		
**Downregulated**		
FKBP11	-0.9516	0.0171
HPN	-0.6668	0.0190
DDOST	-0.8760	0.0349
IDH2	-0.7712	0.0400
**Upregulated**		
GCG	1.5444	0.0002
SMARCA1	0.7268	0.0190
ANKS1B	0.9444	0.0190
MYH10	0.9176	0.0224
PRKAR1A	0.4764	0.0349
CEACAM1	0.7428	0.0349
AGER	0.9520	0.0349
MOB1B	1.0460	0.0349
GC	1.1908	0.0349
PLK2	1.0504	0.0402
PCSK2	0.7060	0.0416
ADGRG2	1.0076	0.0416
DPP4	1.1672	0.0416
GLS	1.0140	0.0417

Table S2. Changes in the expression of *AGER* correlated genes for *GCG*^{hi} vs. *GCG*^{lo} islets (Pearson's *r* = 0.999-1.0; 9228 probe sets). Changes are shown as log fold change, P values are FDR corrected. See Figure 1A for volcano plot visualization.

Gene Symbol	Change	P value
**All donors (23 genes)**		
**Downregulated**		
FKBP11	-0.9516	0.0110
TTLL12	-0.6108	0.0110
HPN	-0.6668	0.0143
PLTP	-0.9200	0.0317
EIF3J	-0.7432	0.0317
MYH9	-0.6788	0.0317
FBXL18	-0.5652	0.0317
LMAN2	-0.7004	0.0410
CLDN3	-0.6148	0.0410
ATF4	-0.5876	0.0410
IDH2	-0.7712	0.0450
**Upregulated**		
ANKS1B	0.9444	0.0143
PRKAR1A	0.4764	0.0317
CEACAM1	0.7428	0.0317
AGER	0.9520	0.0317
GLS	1.0140	0.0317
PTPN13	0.7000	0.0410
RTN1	1.0264	0.0428
PLCB4	1.0060	0.0448
PCSK2	0.7060	0.0450
ADGRG2	0.9824	0.0450
DPP4	1.0452	0.0450
PLK2	1.0504	0.0450

Gene Symbol	Change	P value
**Type 1 diabetes donors (27 genes)**		
**Downregulated**		
KCNQ1	-1.4460	0.0278
PCMTD1	-0.9400	0.0278
HPN	-1.0590	0.0281
SCRIB	-1.0130	0.0320
IDH2	-1.1190	0.0401
FKBP11	-1.2390	0.0445
**Upregulated**		
AGER	1.9690	0.0125
HTATSF1	1.3420	0.0179
SLC22A17	1.6310	0.0179
BCOR	0.9990	0.0278
DPP4	1.1410	0.0278
GATC	1.3540	0.0278
HECW2	1.4050	0.0278
DACH1	1.5140	0.0278
MMP16	1.5450	0.0278
CAMK2N1	1.6560	0.0278
GLS	1.9340	0.0278
ADGRG2	1.8950	0.0281
SMARCA1	2.0810	0.0281
PLK2	1.1430	0.0320
PRKAR1A	0.8030	0.0401
SEZ6L	1.2540	0.0401
CEACAM1	1.3600	0.0401
CXorf57	1.7900	0.0401
EDN3	1.9980	0.0401
SCD	2.0650	0.0409
ANKS1B	1.5930	0.0473

Gene Symbol	Change	P value
**All donors and type 1 diabetes donors only**		
**(11 genes in common)**		
**Downregulated**		
FKBP11	-0.9516	0.0110
HPN	-0.6668	0.0143
IDH2	-0.7712	0.0450
**Upregulated**		
ANKS1B	0.9444	0.0143
PRKAR1A	0.4764	0.0317
CEACAM1	0.7428	0.0317
AGER	0.9520	0.0317
GLS	1.0140	0.0317
ADGRG2	0.9824	0.0450
DPP4	1.0452	0.0450
PLK2	1.0504	0.0450

Table S3. Changes in the expression of *AGER* correlated genes for GCG^{hi} vs. GCG^{lo} islets (Pearson's r = 0.9999-1.0; 3055 probe sets). Changes are shown as log fold change, P values are FDR corrected. See Figure 1A for volcano plot visualization.

Gene Symbol	Change	P value
**All donors (12 genes)**		
**Downregulated**		
HPN	-0.6668	0.0193
EIF3J	-0.7432	0.0230
MYH9	-0.6788	0.0230
CLDN3	-0.6148	0.0280
LMAN2	-0.7004	0.0291
RPLP0	-0.8176	0.0391
**Upregulated**		
AGER	0.9520	0.0230
CEACAM1	0.7428	0.0230
GLS	1.0140	0.0230
DPP4	1.0452	0.0357
PLK2	1.0504	0.0357
ASTN1	0.6732	0.0391

Gene Symbol	Change	P value
**Type 1 diabetes only (9 genes)**		
**Downregulated**		
HPN	-1.0590	0.0306
**Upregulated**		
AGER	1.9690	0.0042
SLC22A17	1.6310	0.0075
GLS	1.9340	0.0231
MMP16	1.5450	0.0238
SEZ6L	1.2540	0.0358
CEACAM1	1.3600	0.0358
CXorf57	1.7900	0.0358
EDN3	1.9980	0.0358

Gene Symbol	Change	P value
**All donors and type 1 diabetes donors only (4 genes in common)**		
**Downregulated**		
HPN	-0.6668	0.0193
**Upregulated**		
AGER	0.9520	0.0230
CEACAM1	0.7428	0.0230
GLS	1.0140	0.0230

Table S4. Changes in the expression of *AGER* correlated genes for GCG^{hi} vs. GCG^{lo} islets (Pearson's *r* = 0.99999-1.0; 1064 probe sets). Changes are shown as log fold change, P values are FDR corrected. See Figure 1A for volcano plot visualization.

Gene Symbol	Change	P value	Gene Symbol	Change	P value	Gene Symbol	Change	P value
**All groups (15 genes)**			**Type 1 diabetes only (5 genes)**			**All donors and type 1 diabetes donors only (2 genes in common)**		
**Downregulated**			**Downregulated**			**Downregulated**		
EIF3J	-0.7432	0.0206	*MUC1*	-1.0230	0.0475	*MUC1*	-0.7664	0.0298
LMAN2	-0.7004	0.0206	**Upregulated**			**Upregulated**		
CLDN3	-0.6148	0.0206	*AGER*	1.9690	0.0015	*AGER*	0.9520	0.0206
CD44	-0.8824	0.0298	*SLC22A17*	1.6310	0.0027			
CFTR	-0.8644	0.0298	*SEZ6L*	1.2540	0.0290			
MUC1	-0.7664	0.0298	*EDN3*	1.9980	0.0290			
PDGFRL	-0.6792	0.0298						
RPL18A	-0.6608	0.0298						
GALNT1	-0.5248	0.0380						
GALNT2	-0.4228	0.0380						
XBP1	-0.7292	0.0473						
**Upregulated**								
AGER	0.9520	0.0206						
DPP4	1.0452	0.0206						
PLK2	1.0504	0.0206						
ASTN1	0.6732	0.0237						

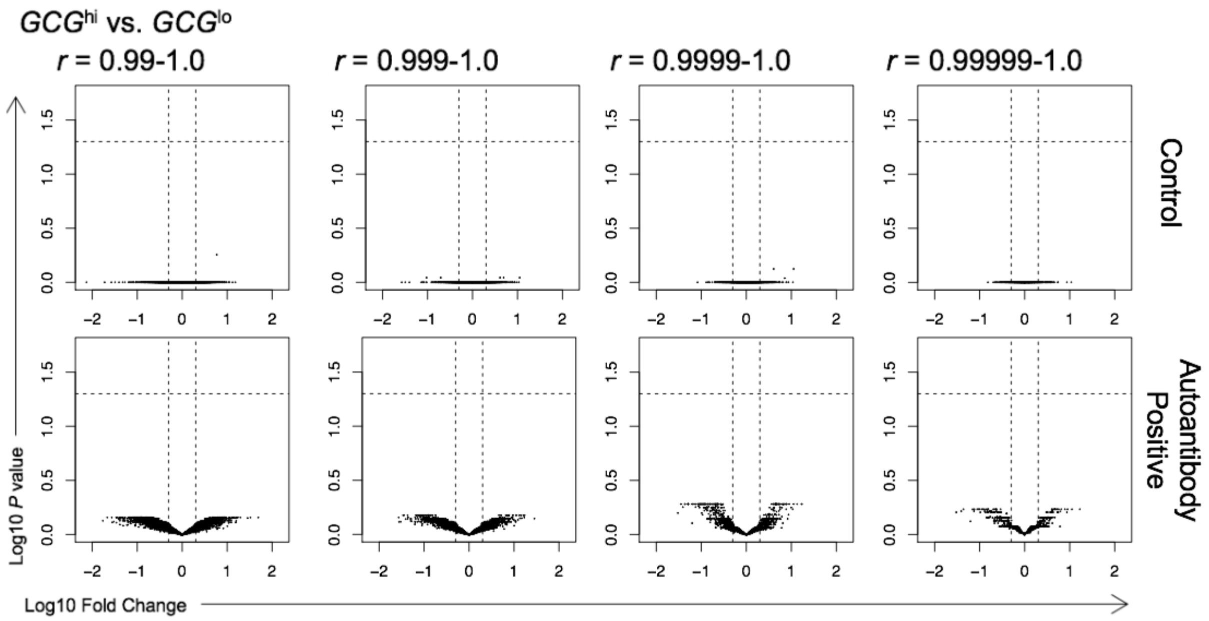

Figure S1. *GCG* expression does not associate with changes in the expression of *AGER* and its correlated genes in the islets of control and non-diabetic autoantibody-positive donors.

Control (n = 18) and non-diabetic autoantibody-positive (n = 12) donors. Significance was defined by FDR q < 0.05, fold-change > 2.0.

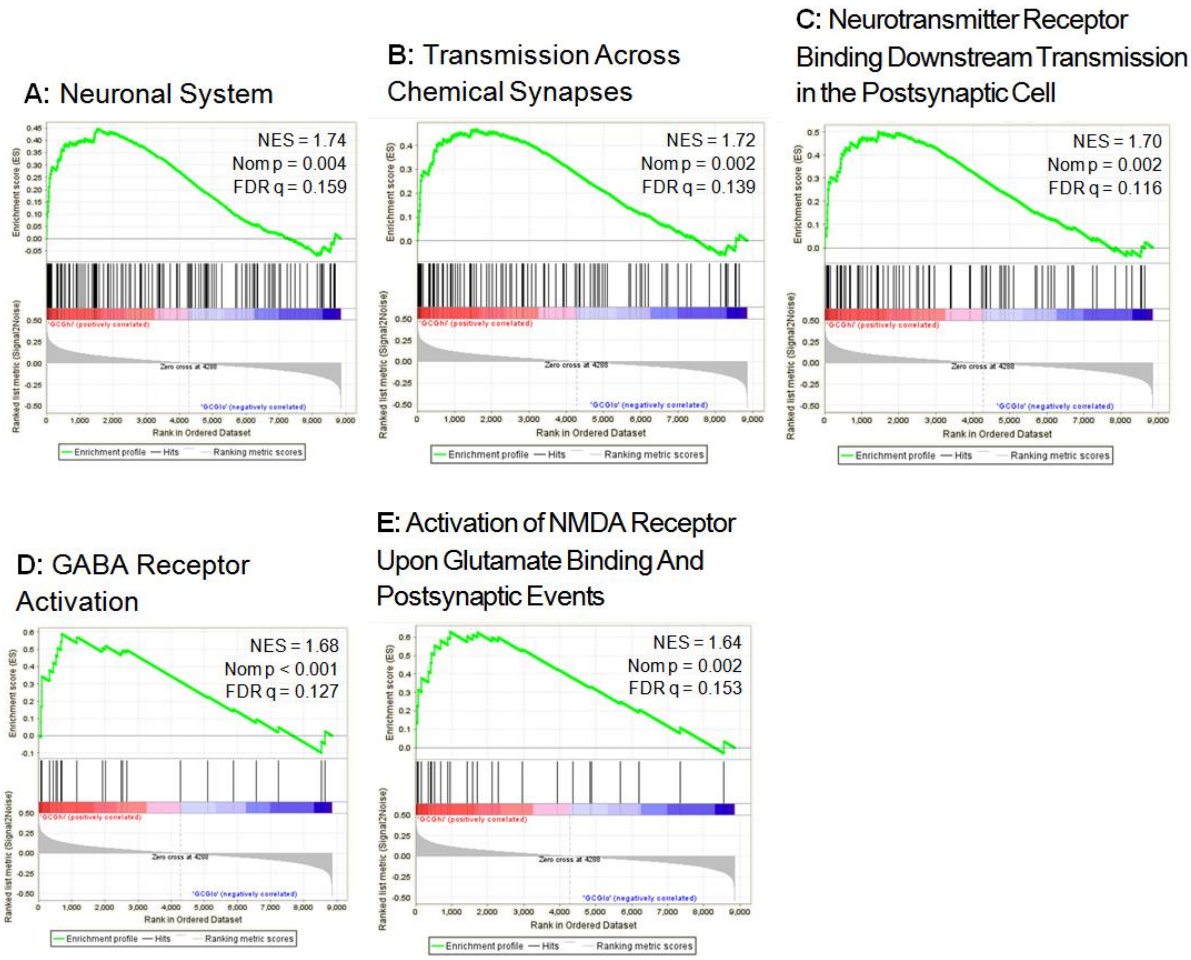

Figure S2. Gene Set Enrichment Analysis (GSEA) using Reactome Pathway Database for *AGER* and its correlated genes in *GCG*^{hi} vs. *GCG*^{lo} islets.

(A-E) GSEA enrichment score curves. NES = normalized enrichment score, Nom p = nominal p value, FDR q = false discovery rate q values. *AGER* and its correlated genes were selected on a Pearson's *r* threshold of 0.99-1.0 (20,937 probe sets).

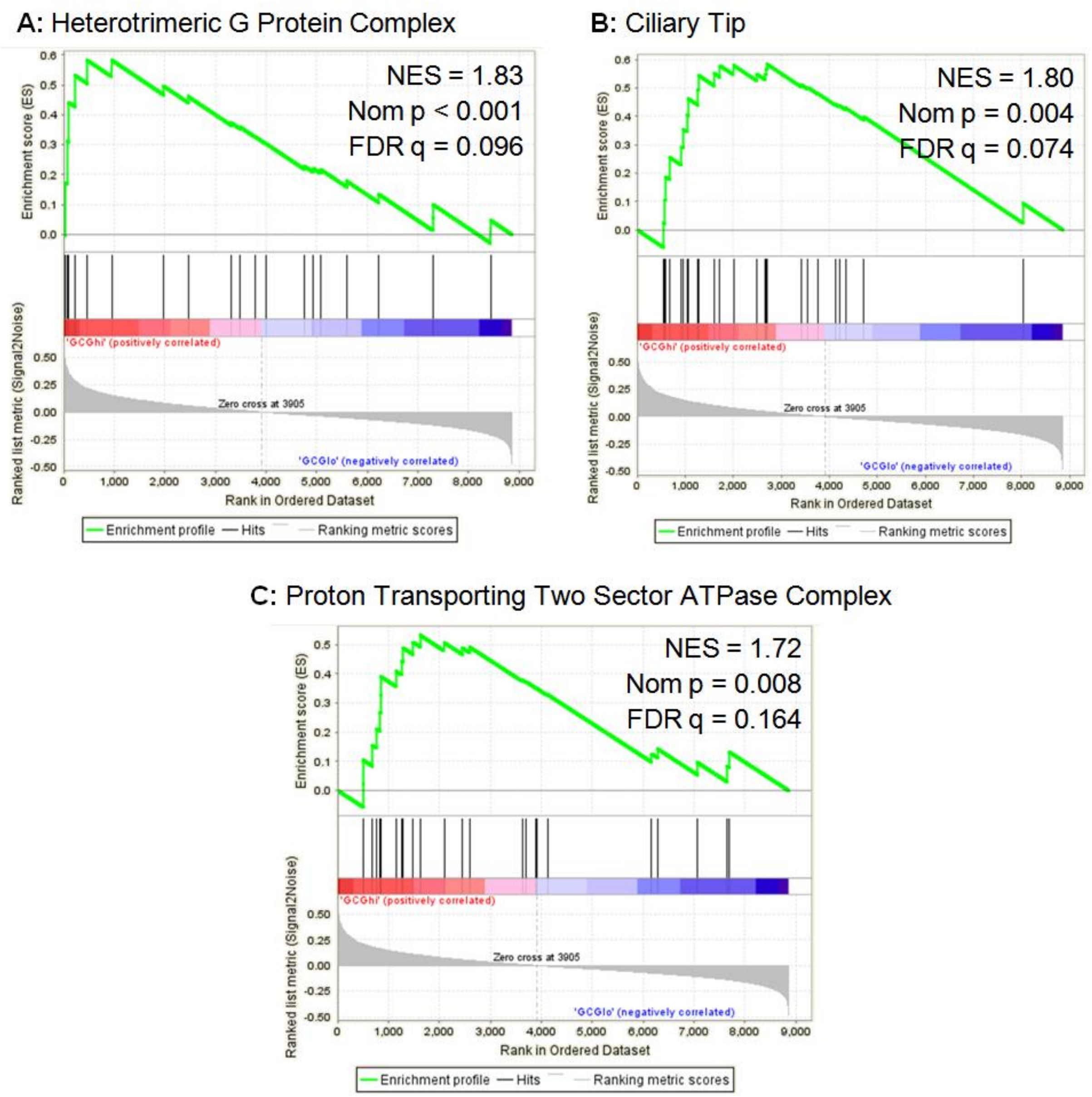

Figure S3. GSEA using Gene Ontology Cellular Components for *AGER* and its correlated genes in *GCG*^{hi} vs. *GCG*^{lo} islets.

(A-E) GSEA enrichment score curves. NES = normalized enrichment score, Nom p = nominal p value, FDR q = false discovery rate q values. *AGER* and its correlated genes were selected on a Pearson's *r* threshold of 0.99-1.0 (20,937 probe sets).

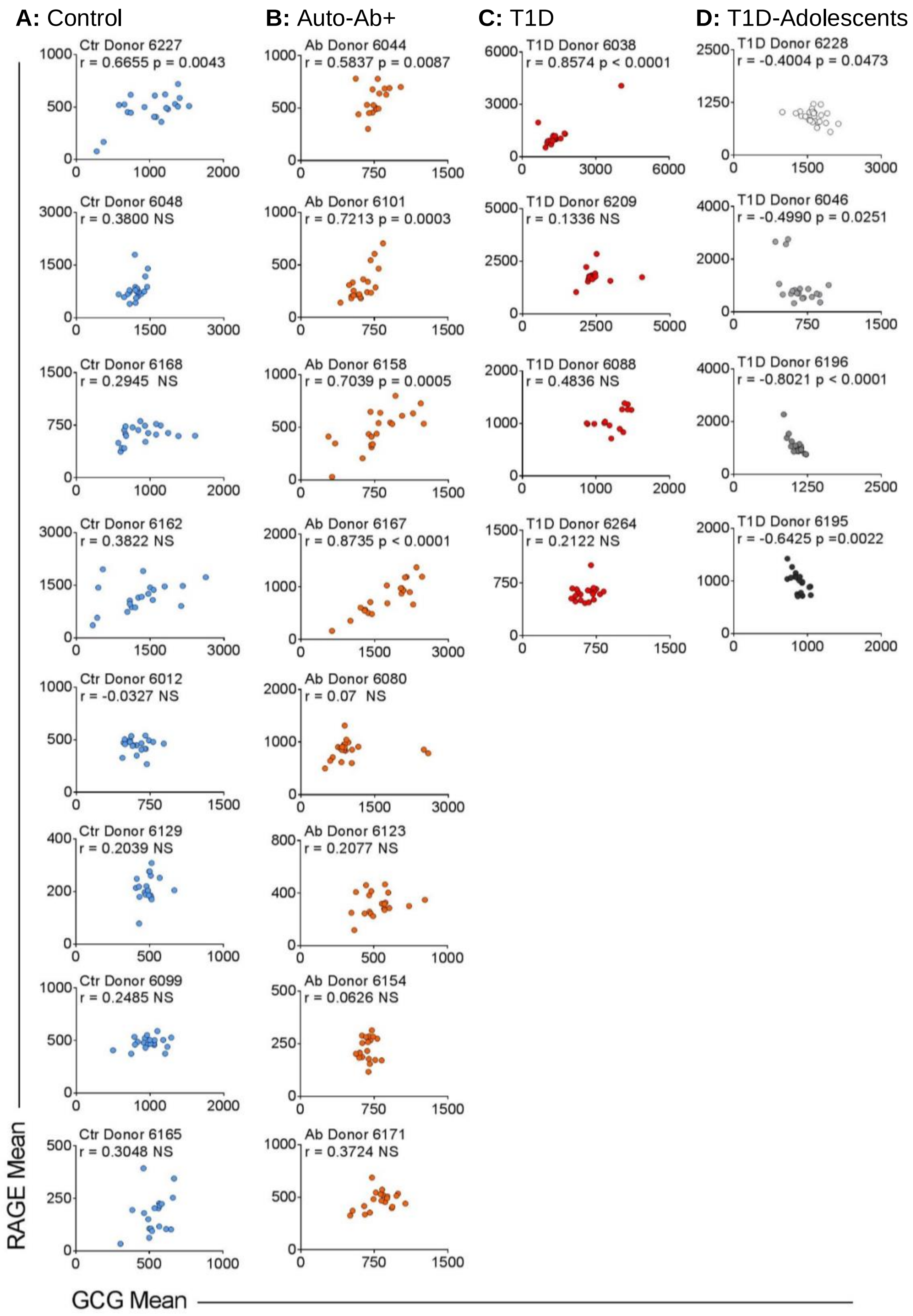

Figure S4. Correlation analyses of glucagon and RAGE expression in the alpha cells for individual donors by quantitative confocal microscopy.

(A-D) Pearson's r for the control (Ctr), non-diabetic autoantibody-positive (Auto-Ab^{+} or Ab) and type 1 diabetes cohorts are shown. P values are indicated in the figure; NS, not significant. Pearson's r for the Ctr, Ab and type 1 diabetes groups overall are in Figure 3H.
